# Fenfluramine Attenuates Retinal Microglial Activation but Does Not Rescue Structural and Vascular Deficits in a Rat Model of Dravet Syndrome

**DOI:** 10.3390/ijms27135752

**Published:** 2026-06-25

**Authors:** Yajuan Zhang, Weixin Qian, Miao Li, Ying-Ying Zou, Zhonghua Lu, Zhihui Huang, Robert K. Naumann, Hong Wang

**Affiliations:** 1 School of Pharmacy, Hangzhou Normal University, Hangzhou 311121, China; 2023112025026@stu.hznu.edu.cn (Y.Z.); huang0069@hznu.edu.cn (Z.H.); 2 The Institute of Biomedical and Health Engineering, Shenzhen Institutes of Advanced Technology, Chinese Academy of Sciences, 1068 Xueyuan Blvd, Shenzhen 230027, China; wx.qian@siat.ac.cn (W.Q.); m.li5@siat.ac.cn (M.L.); zh.lu@siat.ac.cn (Z.L.); 3 Division of Life Sciences and Medicine, University of Science and Technology of China, Hefei 518055, China; 4 Department of Pathology and Pathophysiology, Faculty of Basic Medical Sciences, Kunming Medical University, Kunming 650500, China; zouyingyingzyy@126.com

**Keywords:** Dravet syndrome, fenfluramine, retina, electroretinograph, microglia

## Abstract

Dravet syndrome (DS) is a severe developmental and epileptic encephalopathy caused by *SCN1A* haploinsufficiency. While brain pathology has been extensively studied, the retina remains underexplored. This study investigated retinal structural, functional, vascular, and cellular changes in a *Scn1a*^+/−^ rat model of DS. Anatomical quantification revealed thinning of the retinal nerve fiber layer and thickening of the outer plexiform layer. Electroretinography (ERG) showed selectively reduced oscillatory potential amplitudes, suggesting dysfunction of neurovascular coupling. Consistent with these findings, immunohistochemistry demonstrated aberrant vascular morphology, including increased vessel curvature and reduced branching density. In addition, we observed robust microglial activation in the outer and inner plexiform layers; however, astrocyte morphology remained largely unchanged. Fenfluramine, an approved anti-seizure drug for DS, attenuated microglial activation but failed to rescue retinal structural or vascular deficits, indicating a dissociation between its anti-inflammatory and disease-modifying effects. Our findings suggest that multimodal retinal assessment could serve as a noninvasive biomarker platform for monitoring disease progression and therapeutic response in DS.

## 1. Introduction

Dravet syndrome (DS) is a severe developmental and epileptic encephalopathy (DEE) predominantly caused (>95%) by loss-of-function variants in *SCN1A*, the gene encoding the voltage-gated sodium channel subunit Nav1.1 [1]. The clinical hallmarks of DS include hyperthermia-induced seizures, drug-refractory epilepsy, significant neurodevelopmental delays, and an increased risk of sudden unexpected death in epilepsy (SUDEP) [2]. Seizures in infancy are frequently provoked by elevated body temperatures due to fever, vaccinations, or hot baths [2,3,4,5]. After this initial temperature-sensitive phase, patients develop spontaneous seizures and developmental delay, facing an increased risk of comorbidities and SUDEP [2,6].

Photosensitive seizures are common in DS patients from an early age and may precede cognitive decline [7,8]. A photoparoxysmal EEG response (PPR) is well-documented, and there is a strong association between photosensitivity, frequent myoclonic seizures, and a more severe disease prognosis [9]. However, the potential contribution of structural and functional retinal pathology to this photosensitivity remains unexplored. Recent evidence of glial activation in the retina of a mouse model of DS suggests changes in glial cells could be related to retinal pathology [10].

Current therapeutic strategies for DS primarily reduce seizure burden without targeting the underlying molecular deficits. Novel therapies, such as fenfluramine (FFA) and cannabidiol, have demonstrated remarkable clinical benefits [11,12]. Specifically, multiple clinical trials have shown that FFA significantly reduces seizure frequency while improving executive function and sleep quality [13,14,15,16,17,18]. However, the potential protective effects of FFA on retinal pathology remain unknown.

To investigate retinal structural and functional changes in DS and their response to FFA, we used a well-established rat model of DS carrying a frameshift mutation in the exon 20 of the *Scn1a* gene [19]. This rat model faithfully recapitulates the haploinsufficiency of the *Scn1a* gene, heat-sensitive seizures, and subsequent spontaneous seizures. Electroencephalogram (EEG) characteristics, including “burst-and-suppression” patterns with high delta/theta power, closely mirror the clinical phenotypes of DS patients [19].

The *Scn1a*^+/−^ rat model fulfills established criteria for a preclinical model of DS [20]. We sought to investigate the retinal changes to understand the therapeutic effects of FFA and to explore the potential of retinal metrics as diagnostic or prognostic biomarkers. As a neuroectoderm-derived extension of the central nervous system (CNS) [21,22], the retina frequently mirrors the manifestation of neurological disorders [22,23]—a principle established in Alzheimer’s disease, where retinal abnormalities have been validated as windows to brain pathology [23,24].

Structurally, the retina comprises a highly organized, multilayered structure of neurons, glial cells, and vasculature [25,26]. Visual processing begins when photoreceptors transduce light into neural signals, which are then relayed through intermediate neurons to retinal ganglion cells (RGCs) for integration. The axons of RGCs constitute the nerve fiber layer (NFL) and the optic nerve, ultimately transmitting electrical impulses to the brain [27,28].

Beyond neurons, glial cells—specifically microglia and astrocytes—play critical roles in retinal homeostasis [29,30]. Astrocytes are primarily situated within the NFL, whereas microglia are distributed mainly across the outer plexiform layer (OPL) and the ganglion cell layer (GCL) [31]. As the resident immune sentinels of the retina, microglia become activated in response to injury or stress [30]. However, chronic activation can compromise retinal physiology and is implicated in the pathogenesis of various retinal diseases [32]. Consequently, pharmacological interventions targeting activated microglia represent a promising therapeutic strategy for treating retinal pathologies [30,33].

To assess retinal function, we employed electroretinography (ERG), which captures the retina’s electrical responses to light stimuli. As a sensitive monitor of disease progression in retinal disorders, ERG is particularly effective in detecting early, subclinical functional deficits in epilepsies, which often precede structural changes [34,35].

Here, we assessed retinal structure and function in a rat model of Dravet syndrome and evaluated the therapeutic efficacy of FFA. We hypothesize that DS is associated with retinal structural and functional alterations amenable to pharmacological interventions and that FFA would mitigate these pathological changes. Our objectives are threefold: (1) to characterize retinal morphological and functional deficits in *Scn1a*^+/−^ rats, (2) to determine whether FFA preserves retinal integrity, and (3) to evaluate retinal parameters as non-invasive diagnostic or prognostic biomarkers for DS.

## 2. Results

### 2.1. Thinning of the Retinal Nerve Fiber Layer (NFL) and Thickening of the Outer Plexiform Layer (OPL) in Scn1a^+/−^ Rats

**Figure 1:**
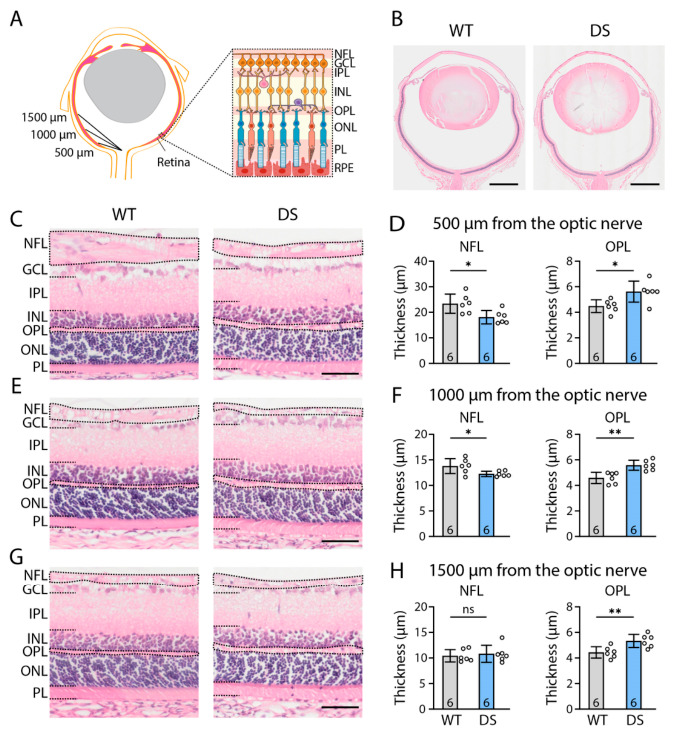
Retinal morphology in wildtype (WT) and *Scn1a*^+/−^ (DS) rats. (**A**) Schematic illustration of ocular and retinal anatomy. Inserts show representative location analyzed: cross-sections 500, 1000, and 1500 µm from the optic disk, corresponding to central, mid-peripheral, and peripheral retina. (**B**) Representative H&E-stained whole-eye sections from WT and *Scn1a*^+/−^ (DS) rats. Scale bar = 1 mm. (**C**,**E**,**G**) High-magnification images of retinal cross-sections at 500 µm (**C**), 1000 µm (**E**), and 1500 µm (**G**) from the optic disk. Black dotted areas indicate the nerve fiber layer (NFL) and outer plexiform layer (OPL). Scale bars: 50 µm. (**D**,**F**,**H**) Quantification of NFL and OPL thickness at 500 µm (**D**), 1000 µm (**F**), and 1500 µm (**H**) from the optic nerve. Data are presented as mean ± SD; n = 6 rats per group. Statistical significance was assessed by an unpaired two-tailed *t*-test: At 500 µm: NFL, *p* = 0.0181; OPL, *p* = 0.0157; At 1000 µm: NFL, *p* = 0.0359; OPL, *p* = 0.0021; At 1500 µm: NFL, *p* = 0.65; OPL, *p* = 0.0091. Significance levels: *p* < 0.05 (*), *p* < 0.01 (**), non-significant (ns).

To evaluate the impact of the *Scn1a* mutation on retinal architecture, we performed hematoxylin and eosin (H&E) staining on cross-sections of eyes of postnatal day 30 (P30) *Scn1a*^+/−^ (DS) rats and their wild-type (WT) littermates. We analyzed retinal thickness at three distinct positions: 500 µm, 1000 µm, and 1500 µm from the optic disk (Figure 1A,B). Overall, global ocular morphology appeared comparable between the two genotypes (Figure 1B). However, detailed morphometric analysis revealed significant layer-specific alterations.

**Figure A1:**
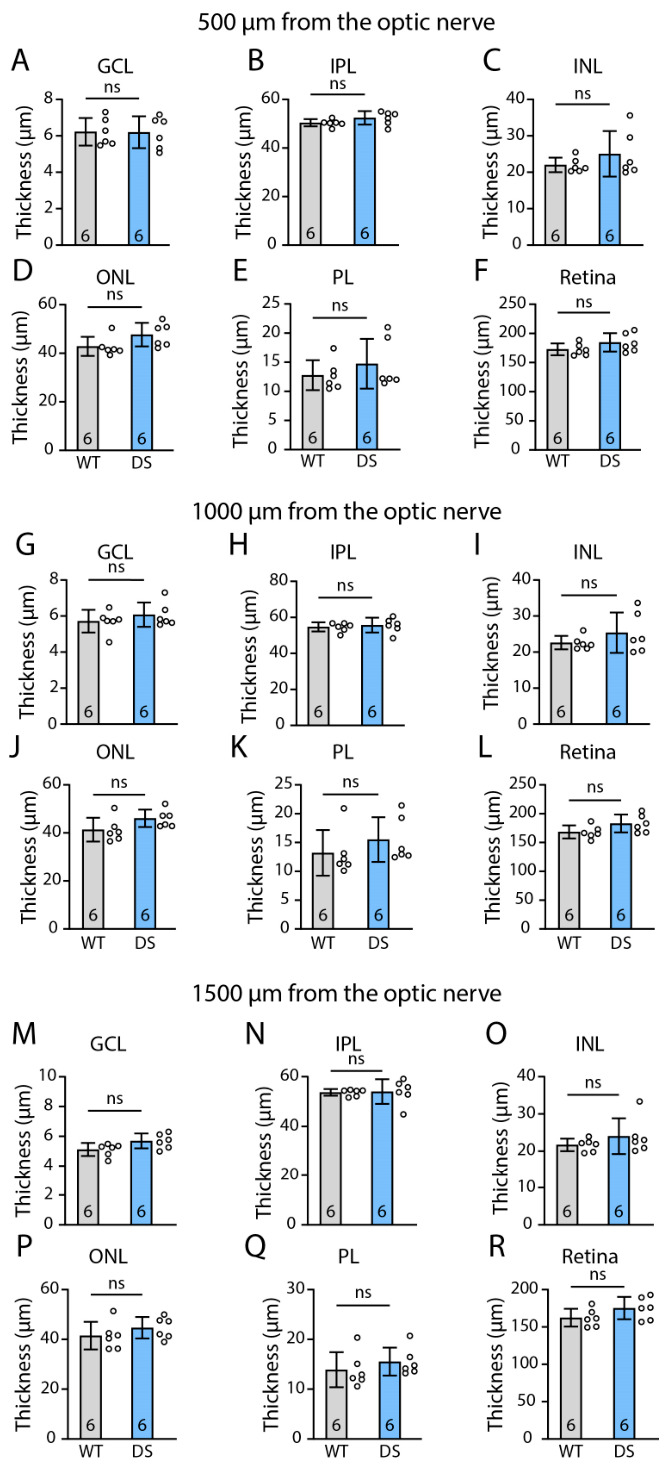
Retinal morphology in wild-type (WT) and *Scn1a*^+/−^ (DS) rats. (**A**–**F**) Quantitative analysis of the thickness of the ganglion cell layer (GCL) (**A**), inner plexiform layer (IPL) (**B**), inner nuclear layer (INL) (**C**), outer nuclear layer (ONL) (**D**), photoreceptor layer (PL) (**E**), and retina from NFL-PL (**F**) at 500 µm from the optic nerve. Data are presented as Mean ± SD, n = 6 rats per group. unpaired *t*-test: GCL *p* = 0.9593; IPL *p* = 0.1482; INL *p* = 0.2821; Retina *p* = 0.1513. Mann–Whitney test: ONL *p* = 0.0649; PL *p* = 0.3939. (**G**–**L**) Quantitative analysis of the thickness of GCL (**G**), IPL (**H**), INL (**I**), ONL (**J**), PL (**K**), and retina from NFL-PL (**L**) at 1000 µm from the optic nerve. Data are presented as Mean ± SD, n = 6 rats per group. unpaired *t*-test: GCL *p* = 0.3607; IPL *p* = 0.6305; INL *p* = 0.2778; ONL *p* = 0.0893; Retina *p* = 0.0907. Mann–Whitney test: PL *p* = 0.1320. (**M**–**R**) Quantitative analysis of the thickness of GCL (**M**), IPL (**N**), INL (**O**), ONL (**P**), PL (**Q**), and retina from NFL-PL (**R**) at 1500 µm from the optic nerve. Data are presented as Mean ± SD, n = 6 rats per group. unpaired *t*-test: GCL *p* = 0.0574; ONL *p* = 0.2987; PL *p* = 0.3961; Retina *p* = 0.1309. Mann–Whitney test: IPL *p* = 0.5887, INL *p* = 0.3095. Significance levels: non-significant (ns).

We observed a significant reduction in the thickness of the nerve fiber layer (NFL) in DS rats compared to WT controls at 500 µm and 1000 µm sampled locations (Figure 1C–F). However, the difference did not reach statistical significance at the 1500 µm position (Figure 1G–H). Since the NFL consists of retinal ganglion cell (RGC) axons, this thinning suggests compromised RGC integrity. This finding is consistent with observations in patients with drug-resistant epilepsies [36]. Conversely, we detected significant thickening of the outer plexiform layer (OPL), the synaptic zone connecting photoreceptor terminals to bipolar and horizontal cell dendrites, in *Scn1a*^+/−^ rats (Figure 1C,E,G). Notably, the thickness of the intervening nuclear and plexiform layers (RGC, IPL, INL, and ONL) did not differ significantly between genotypes (Figure A1). Despite these internal rearrangements, the total retinal thickness (measured from the photoreceptor layer to the NFL) remained unchanged between WT and DS rats (Figure A1), suggesting a redistribution of tissue rather than atrophy or hypertrophy.

### 2.2. Electroretinogram Abnormality in Scn1a^+/−^ Rats

**Figure 2:**
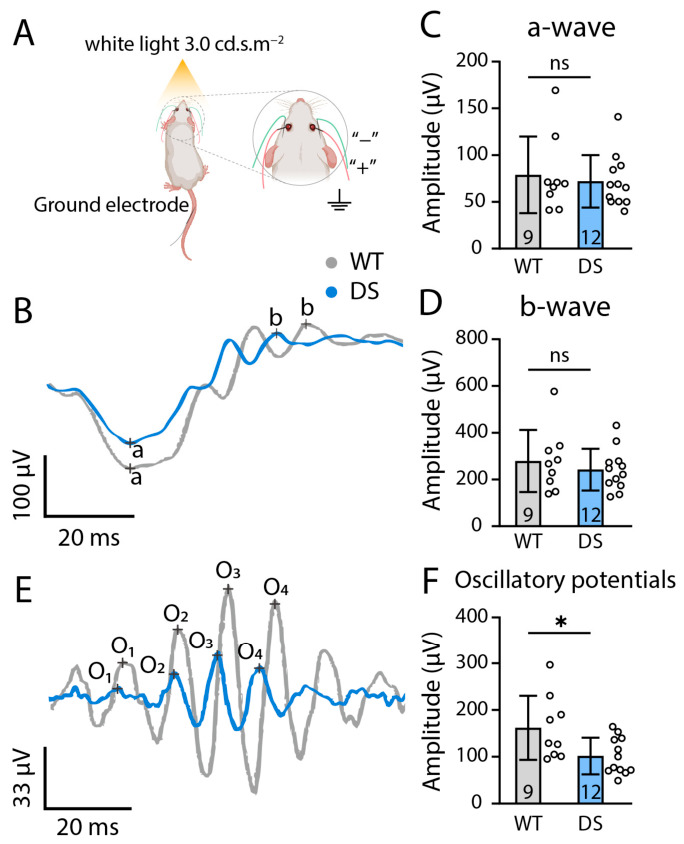
Reduced amplitude of oscillatory potentials in scotopic flash electroretinogram in *Scn1a*^+/−^ (DS) rats. (**A**) Schematic illustration of the electroretinogram (ERG) recording. Recording electrodes were placed on the corneal surface, reference electrodes were inserted subcutaneously, and the ground electrode was placed in the tail. Rats were dark-adapted overnight prior to white light stimulation with a time-integrated luminance of 3.0 cd·s·m^−2^. (**B**) Representative traces of maximal mixed (scotopic) ERG responses from wild-type (WT) and DS rats. (**C**,**D**) Quantification of a-wave (**C**) and b-wave (**D**) amplitudes.The a-wave represents the amplitude at the trough, and the b-wave represents the amplitude difference from point a to point b. Data are presented as mean ± SD, with n = 9 rats for WT and n = 12 rats for DS. Mann-Whitney and unpaired *t*-tests, *p* = 0.70 for (**C**) and *p* = 0.45 for (**D**). (**E**) Representative traces of scotopic ERG recordings highlighting oscillatory potentials from WT and DS rats. (**F**) Amplitudes of oscillatory potentials are quantified as the summed peak-to-trough responses. Data are expressed as mean ± SD, with n = 9 rats for WT and n = 12 rats for DS. Unpaired *t*-test, *p* = 0.0196. Significance levels: *p* < 0.05 (*), non-significant (ns).

**Figure A2:**
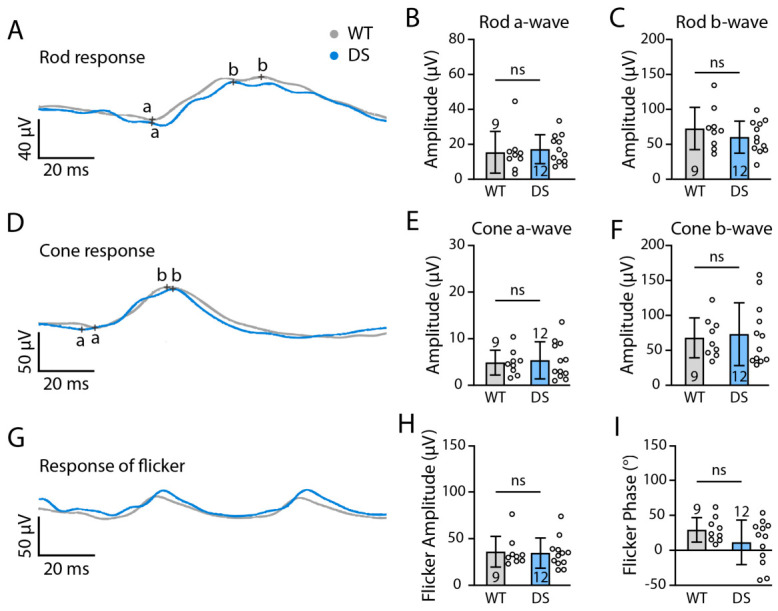
Flash electroretinograms (FERG) of *Scn1a*^+/−^ (DS) and wild-type rats. Different lowercase letters indicate significant differences between groups (*p* < 0.05) (**A**) Dark-adapted rod responses (Rod) to flashes of white light at an intensity of 0.015 cd.s.m^−2^ in wild-type and DS rats. (**B**,**C**) Quantification of the amplitudes of Rod-a (**B**) and Rod-b (**C**). Data are presented as mean ± SD, with n = 9 rats for WT and n = 12 rats for DS. Mann–Whitney test: *p* = 0.4745 for (**B**); Unpaired *t*-test *p* = 0.3014 for (**C**). (**D**) Light-adapted cone responses (Cone) to flashes of white light at an intensity of 3.0 cd.s.m^−2^ in wild-type and DS rats. (**E**,**F**) Quantification of the amplitudes of Cone-a (**E**) and Cone-b (**F**). Data are presented as mean ± SD, with n = 9 rats for WT and n = 12 rats for DS. Unpaired *t*-test: *p* = 0.7579 for (**E**); Mann–Whitney test: *p* = 0.9170 for (F). (**G**) Light-adapted 20 Hz Flicker responses to flashes of white light at an intensity of 3.0 cd.s.m^−2^ in wild-type and DS rats. (**H**,**I**) Quantification of Flicker amplitude (**H**) and Flicker phase (**I**). Data are presented as mean ± SD, with n = 9 rats for WT and n = 12 rats for DS. Mann–Whitney test: *p* = 0.9867 for (**H**); Unpaired *t*-test: *p* = 0.1481 for (**I**). Significance levels: non-significant (ns).

To determine whether the observed retinal structural alterations resulted in functional deficits, we performed full-field electroretinography (ERG) to assess light-evoked retinal responses (Figure 2A). Under dark-adapted (scotopic) conditions, both wild-type (WT) and *Scn1a*^+/−^ rats demonstrated well-defined a- and b-waves in the maximal mixed rod-cone response (Figure 2B). Quantitative analysis revealed no significant differences in a- or b-wave amplitudes or implicit times between *Scn1a*^+/−^ and WT littermates (Figure 2C,D). Consistent with these findings, isolated scotopic rod responses (Figure A2A–C) and photopic cone responses (Figure A2D–F) also showed comparable waveform parameters between genotypes. Furthermore, 20 Hz flicker ERG recordings revealed no differences in response amplitude or phase, indicating preserved cone pathway function in *Scn1a*^+/−^ rats (Figure A2G–I).

Since outer retinal signaling appeared intact, we evaluated inner retinal microcircuit activity by extracting oscillatory potentials (OPs) from the scotopic maximal response (Figure 2E). High-pass filtering isolated a series of high-frequency wavelets superimposed on the rising phase of the b-wave. Notably, *Scn1a*^+/−^ rats exhibited a marked reduction in OP amplitude compared to WT littermates (Figure 2F). OPs are well-established electrophysiological markers of inner retinal synaptic integrity and have been closely linked to retinal neurovascular coupling [37]. Therefore, the specific attenuation of OPs in *Scn1a*^+/−^ rats suggests a selective impairment in retinal neurovascular communication independent of primary photoreceptor or bipolar cell dysfunction.

### 2.3. Fenfluramine Treatment Fails to Rescue Retinal Structural Abnormalities in Scn1a^+/−^ Rats

**Figure 3:**
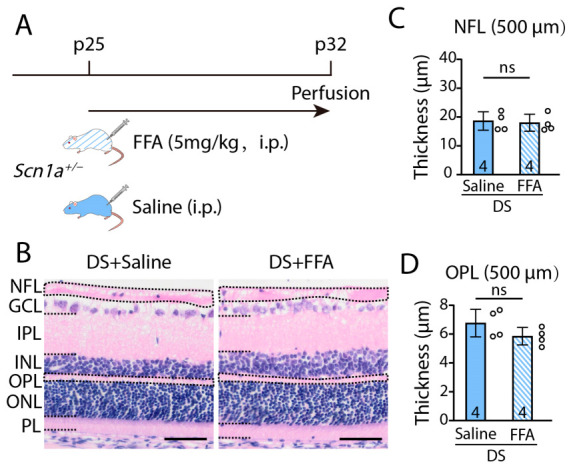
Fenfluramine (FFA) does not alter retinal structure in *Scn1a*^+/−^ (DS) rats. (**A**) Schematic of the FFA treatment regimen. DS rats were divided into two groups. One group received daily intraperitoneal injections of 5 mg/kg FFA (treatment group) starting from P25 to P32, while the control group received saline (vehicle). (**B**) Representative H&E-stained retinal cross-section at 500 µm from the optic nerve in DS rats treated with saline or FFA. Scale bar = 50 µm. (**C**,**D**) Quantification of retinal layer thickness in the nerve fiber layer (NFL) (**C**) and outer plexiform layer (**D**) at 500 µm from the optic nerve. Data presented as mean ± SD; n = 4 rats per group. Unpaired *t*-test, *p* = 0.79 for (**C**) and *p* = 0.1584 for (**D**). Significance levels: non-significant (ns).

**Figure A3:**
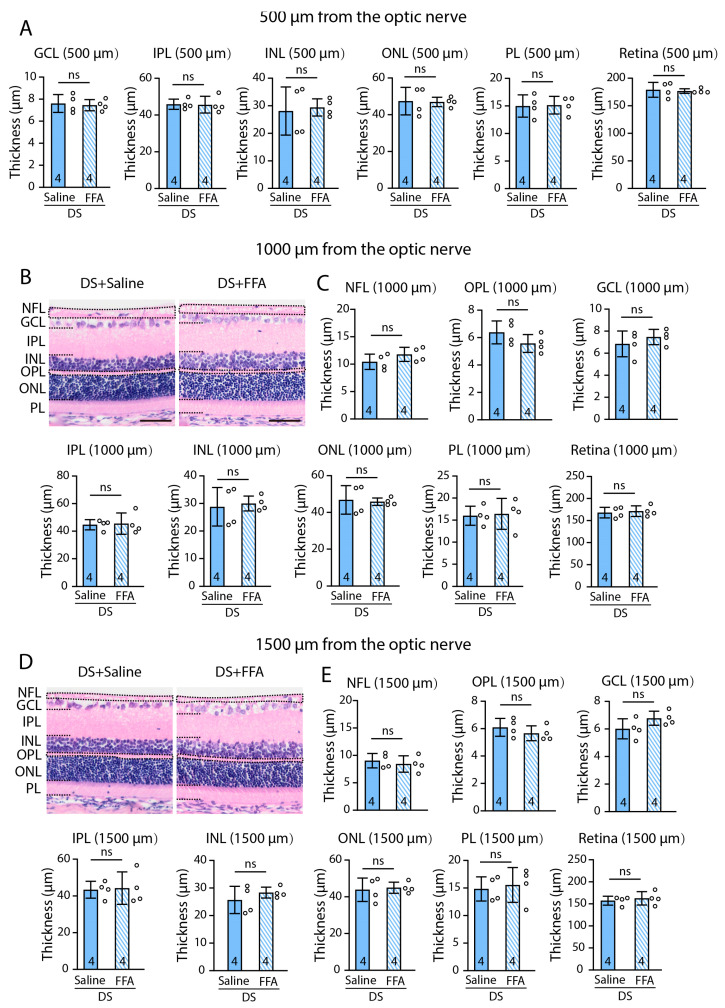
Retinal morphology of *Scn1a*^+/−^ (DS) rats treated with fenfluramine and saline. (**A**) Quantitative analysis of the thickness of the ganglion cell layer (GCL), inner plexiform layer (IPL), inner nuclear layer (INL), outer nuclear layer (ONL), photoreceptor layer (PL), and retina from NFL-PL at 500 µm from the optic nerve. Data are presented as Mean ± SD, n = 4 rats per group. unpaired *t*-test: GCL *p* = 0.7613; IPL *p* = 0.9389; ONL *p* = 0.9133; PL *p* = 0.9059. Mann–Whitney test: INL *p* > 0.9999; Retina *p* > 0.9999. (**B**,**D**) Representative HE-stained retinal cross-section at 1000 µm (**B**) and 1500 µm (**D**) from the optic nerve in DS rats treated with saline or FFA. Scale bar = 50 µm. (**C**) Quantitative analysis of the thickness of the NFL, OPL, GCL, IPL, INL, ONL, PL, and retina from NFL-PL at 1000 µm from the optic nerve. Data are presented as Mean ± SD, n = 4 rats per group. unpaired *t*-test: NFL *p* = 0.2042; OPL *p* = 0.1783; GCL *p* = 0.3955; IPL *p* = 0.8541; INL *p* = 0.7615; ONL *p* = 0.816; PL *p* = 0.8453; Retina *p* = 0.7141. (**E**) Quantitative analysis of the thickness of the NFL, OPL, GCL, IPL, INL, ONL, PL, and retina from NFL-PL at 1500 µm from the optic nerve. Data are presented as Mean ± SD, n = 4 rats per group. unpaired *t*-test: NFL *p* = 0.567; OPL *p* = 0.3471; GCL *p* = 0.1357; IPL *p* = 0.8686; INL *p* = 0.3506; ONL *p* = 0.7648; PL *p* = 0.723. Mann–Whitney test: Retina *p* = 0.8857. Significance levels: non-significant (ns).

Fenfluramine (FFA) is a clinically approved antiseizure medication that effectively reduces seizure frequency in patients with Dravet syndrome [13,14,15]. However, whether its therapeutic benefits extend to retinal pathology remains unknown. To address this, we administered FFA daily to *Scn1a*^+/−^ rats from P25 to P32 and evaluated retinal morphology as an indicator of potential neuroprotective effects (Figure 3A). Quantitative analysis of retinal layer thickness revealed that FFA treatment did not significantly alter nerve fiber layer (NFL) or outer plexiform layer (OPL) dimensions compared to vehicle-treated *Scn1a*^+/−^ controls (Figure 3B–D, at 500 µm from the optic disk, Figure A3; 1000 µm and 1500 µm from the optic disk).

Collectively, these findings indicate that 8-day FFA treatment fails to reverse the characteristic NFL thinning and OPL widening in *Scn1a*^+/−^ rats, suggesting limited direct structural protection to the retina under these conditions.

### 2.4. Fenfluramine Attenuates Microglial Activation in the Retina of Scn1a^+/−^ Rats

**Figure 4:**
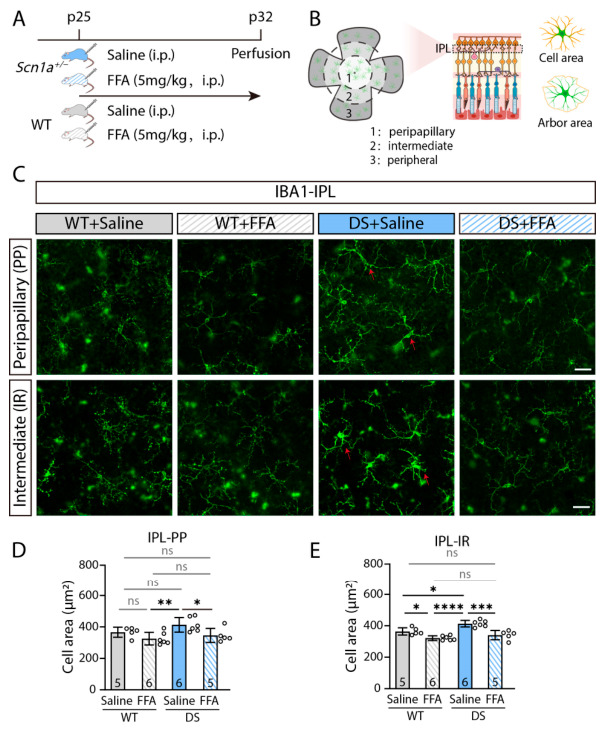
Fenfluramine (FFA) alleviates overactivation of IPL microglia in *Scn1a*^+/−^ (DS) rats. (**A**) Schematic of FFA treatment regime. WT and DS rats were divided into two groups: one received daily intraperitoneal injections of 5 mg/kg fenfluramine (treatment group) starting from P25 to P32, while the control group received saline (vehicle). (**B**) Schematic illustration of the regional segmentation used for IPL microglia analysis: peripapillary (PP), intermediate (IR), and peripheral (PR) regions. Cell area refers to the area of each microglial cell. The arbor area refers to the area formed by the connections of microglial processes. (**C**) Representative Iba1 immunofluorescence labeling showing microglia in the IPL. Red arrows indicate enlarged somata, suggestive of microglia overactivation. Scale bar = 25 µm. (**D**,**E**) Quantification of glial cell body area in the peripapillary (**D**) and intermediate (**E**) regions. FFA treatment rescued microglial hypertrophy in DS rats, indicating reduced neuroinflammatory signaling in the retina. Mean ± SD; per group: WT + saline n = 5, WT + FFA n = 6, DS + saline n = 6, DS + FFA n = 5. Two-way ANOVA: WT + FFA vs. DS + saline *p* = 0.003; DS + saline vs. DS + FFA *p* = 0.0268 (**D**). WT + saline vs. DS + saline *p* = 0.0103; WT + FFA vs. DS + saline *p* < 0.0001; DS + saline vs. DS + FFA *p* = 0.0003 (**E**). Significance levels: *p* < 0.05 (*), *p* < 0.01 (**), *p* < 0.001 (***), *p* < 0.0001 (****), non-significant (ns).

Although fenfluramine (FFA) did not rescue gross structural abnormalities, we investigated its potential therapeutic effects on retinal cellular morphology. We established four experimental groups using WT and *Scn1a*^+/−^ rats, each subjected to daily FFA or saline treatment (Figure 4A). Since glial overactivation is a hallmark of Dravet syndrome models [10], we first characterized microglial morphology in whole-mount retinas.

We examined microglia within the inner plexiform layer (IPL), which we subdivided into peripapillary (PP), intermediate (IR), and peripheral (PR) regions (Figure 4B). In WT rats, regardless of treatment, Iba1-immunoreactive microglia exhibited a resting phenotype characterized by small somata and long, thin, ramified processes with a high arborization complexity (Figure 4C). Conversely, microglia in saline-treated *Scn1a*^+/−^ rats displayed signs of activation, including enlarged somata, dense Iba1 immunoreactivity, and retracted processes. Notably, FFA administration restored the “resting” morphology in *Scn1a*^+/−^ rats, particularly in the PP and IR regions of the IPL (Figure 4C).

**Figure A4:**
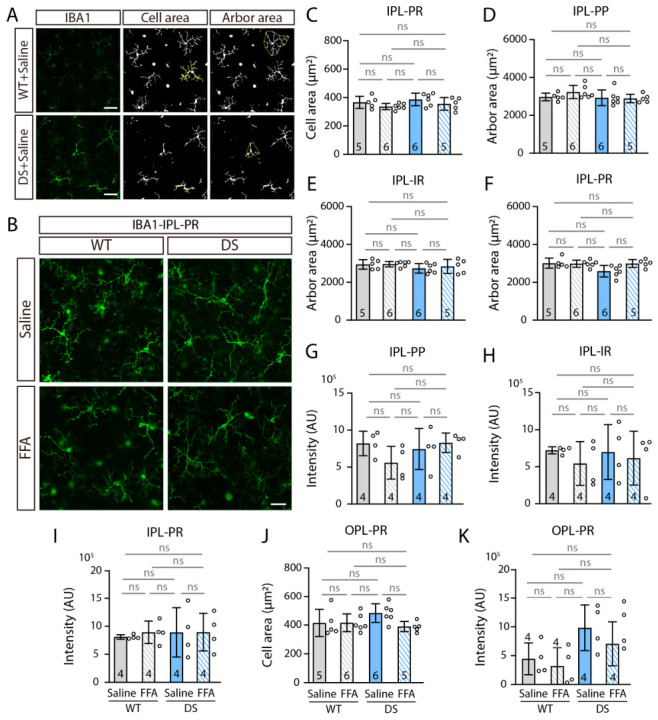
Microglia in the IPL and OPL of the *Scn1a*^+/−^ (DS) and wild-type rat retinas treated and untreated with Fenfluramine (FFA). (**A**) Representative images of microglial cell area and arbor area analyzed by ImageJ 1.8.0 for the WT + Saline and DS + Saline groups. Scale bar = 50 µm. (**B**) Microglia in the IPL Peripheral of retinal whole mounts labeled with Iba1, magnified 40×. Scale bar = 25 µm. (**C**) Quantification of microglial cell area in the IPL Peripheral region. Mean ± SD, number of rats per group: WT + Saline n = 5, WT + FFA n = 6, DS + Saline n = 6, DS + FFA n = 5. Two-way ANOVA, WT + Saline vs. DS + Saline *p* = 0.4989; WT + FFA vs. DS + Saline *p* = 0.0955; DS + Saline vs. DS + FFA *p* = 0.2616. (**D**–**F**) Quantification of microglial arbor area in three regions: IPL peripapillary (**D**), intermediate (**E**), and peripheral (**F**) regions. Mean ± SD, number of rats per group: WT + Saline n = 5, WT + FFA n = 6, DS + Saline n = 6, DS + FFA n = 5. Two-way ANOVA, All values *p* > 0.05, WT + Saline vs. DS + Saline *p* = 0.9391; WT + FFA vs. DS + Saline *p* = 0.3216; DS + Saline vs. DS + FFA *p* = 0.9995 (**C**). WT + Saline vs. DS + Saline *p* = 0.3833; WT + FFA vs. DS + Saline *p* = 0.3996; DS + Saline vs. DS + FFA *p* = 0.7779 (**D**). WT + Saline vs. DS + Saline *p* = 0.0509; WT + FFA vs. DS + Saline *p* = 0.0662; DS + Saline vs. DS + FFA *p* = 0.06 (**E**). (**G**–**I**) Quantification of total fluorescence intensity of microglia in the IPL peripapillary (**G**), intermediate (**H**), and peripheral (**I**) regions. Mean ± SD, n = 4 rats per group. Two-way ANOVA: WT + Saline vs. DS + Saline *p* = 0.9553; WT + FFA vs. DS + Saline *p* = 0.6176; DS + Saline vs. DS + FFA *p* = 0.9382 (**G**). WT + Saline vs. DS + Saline *p* = 0.999; WT + FFA vs. DS + Saline *p* = 0.788; DS + Saline vs. DS + FFA *p* = 0.9575 (**H**). WT + Saline vs. DS + Saline *p* = 0.9789; WT + FFA vs. DS + Saline *p* > 0.9999; DS + Saline vs. DS + FFA *p* > 0.9999 (**I**). (**J**) Quantification of cell area of microglia in the OPL Peripheral region. Mean ± SD, n = 5 for WT + Saline, n = 6 for WT + FFA, n = 6 for DS + Saline, n = 5 for DS + FFA. Two-way ANOVA, WT + Saline vs. DS + Saline *p* = 0.2085; WT + FFA vs. DS + Saline *p* = 0.2869; DS + Saline vs. DS + FFA *p* = 0.0723. (**K**) Quantification of total fluorescence intensity of microglia in the OPL Peripheral region. Mean ± SD, each experimental group animals n = 4. Two-way ANOVA, WT + saline vs. DS + saline *p* = 0.127; WT + FFA vs. DS + saline *p* = 0.0536; DS + saline vs. DS + FFA *p* = 0.5911. Significance levels: non-significant (ns).

To quantify these morphological changes, we measured microglial soma area and arbor area (Figure 4B, Figure A4A). The soma area was significantly larger in *Scn1a*^+/−^ rats than in WT controls, indicating microglial activation in DS rats. Soma area was reduced to WT levels following FFA treatment in the PP and IR regions (Figure 4D,E). However, this protective effect was not observed in the peripheral IPL, where soma area differences between groups remained unchanged (Figure A4C). Furthermore, microglial arbor area remained unaffected across all IPL regions, regardless of genotype or treatment status (Figure A4D–F). We also quantified the Iba1 fluorescent intensity in the IPL and discovered comparable intensity across groups and regions of the IPL (Figure A4G–I).

**Figure 5:**
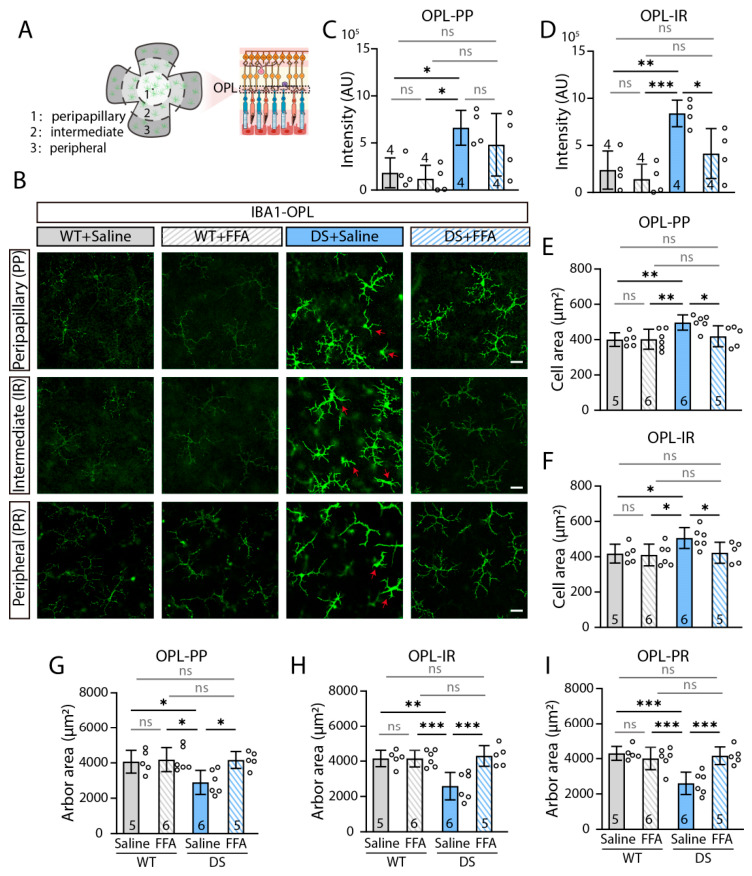
Fenfluramine (FFA) alleviates overactivation of OPL microglia in *Scn1a*^+/−^ (DS) rats. (**A**) Schematic illustration of the regional segmentation used for IPL microglia analysis: peripapillary (PP), intermediate (IR), and peripheral (PR) regions. (**B**) Representative Iba1 immunofluorescence labeling showing microglia in the OPL. Red arrows indicate enlarged somata and the retraction of processes, suggestive of microglia overactivation. Scale bar = 25 µm. (**C**,**D**) Quantification of total fluorescence intensity in Iba1-labeled microglia in the peripapillary (**C**) and intermediate (**D**) regions. FFA treatment reduced fluorescence intensity. Mean ± SD, n = 4 rats per group. Two-way ANOVA: WT + saline vs. DS + saline *p* = 0.0295; WT + FFA vs. DS + saline *p* = 0.015 (**C**). WT + saline vs. DS + saline *p* = 0.0013; WT + FFA vs. DS + saline *p* = 0.0004; DS + saline vs. DS + FFA *p* = 0.0122 (**D**). (**E**,**F**) Quantification of glial cell body area in the peripapillary (**E**) and intermediate (**F**) regions. FFA treatment rescued microglial hypertrophy in DS rats, indicating reduced neuroinflammatory signaling in the retina. (**G**–**I**) Quantification of glial arbor area in the peripapillary (**G**), intermediate (**H**), and peripheral (**I**) regions. FFA treatment rescued the retraction of microglial processes in DS rats, indicating a reduction in neuroinflammatory signaling in the retina. Data are presented as mean ± SD, per group: WT + saline n = 5, WT + FFA n = 6, DS + saline n = 6, DS + FFA n = 5. Two-way ANOVA: WT + Saline vs. DS + Saline *p* = 0.0041; WT + FFA vs. DS + Saline *p* = 0.0059; DS + Saline vs. DS + FFA *p* = 0.0164 (**E**). WT + Saline vs. DS + Saline *p* = 0.025; WT + FFA vs. DS + Saline *p* = 0.0211; DS + Saline vs. DS + FFA *p* = 0.0337 (**F**). WT + Saline vs. DS + Saline *p* = 0.0448; WT + FFA vs. DS + Saline *p* = 0.0109; DS + Saline vs. DS + FFA *p* = 0.0276 (**G**). WT + Saline vs. DS + Saline *p* = 0.0015; WT + FFA vs. DS + Saline *p* = 0.0006; DS + Saline vs. DS + FFA *p* = 0.0007 (**H**). WT + Saline vs. DS + Saline *p* = 0.0003; WT + FFA vs. DS + Saline *p* = 0.0008; DS + Saline vs. DS + FFA *p* = 0.0006 (**I**). Significance levels: *p* < 0.05 (*), *p* < 0.01 (**), *p* < 0.001 (***), non-significant (ns).

Given that H&E staining revealed thickening of the outer plexiform layer (OPL) in *Scn1a*^+/−^ retinas, we next assessed microglial morphology in three OPL subregions (Figure 5A). Similarly to the IPL, microglia in untreated *Scn1a*^+/−^ rats exhibited activated phenotypes, including enlarged somata, process retraction, and robust Iba1 immunoreactivity. FFA treatment effectively rescued these features, restoring soma size and Iba1 intensity to levels comparable to WT controls (Figure 5B).

Quantitative analysis of Iba1 fluorescence intensity confirmed a significant increase in the *Scn1a*^+/−^ group compared to WT in both PP and IR regions (Figure 5C,D and Figure A4K). FFA treatment successfully reversed this increase in the IR region, although differences in the PP region persisted (Figure 5C,D; Figure A4K). Consistent with findings in the IPL, microglial soma area was enlarged in *Scn1a*^+/−^ rats and normalized by FFA treatment in the PP and IR regions (Figure 5E,F), while remaining unchanged in the peripheral region (Figure A4J).

In contrast to the IPL, where arbor area was stable, microglial arbor area in the OPL of saline-treated *Scn1a*^+/−^ rats was significantly reduced across all subregions (Figure 5G–I). Strikingly, FFA treatment fully restored arbor area to WT levels in all OPL regions (Figure 5G–I). Collectively, these data demonstrate that FFA exerts a robust protective effect in the OPL of *Scn1a*^+/−^ rats, evidenced by the complete rescue of microglial arbor area and near-complete normalization of soma size and Iba1 intensity.

### 2.5. Astrocyte Morphology and Distribution Remain Unchanged in Scn1a^+/−^ Rats Regardless of Treatment

**Figure 6:**
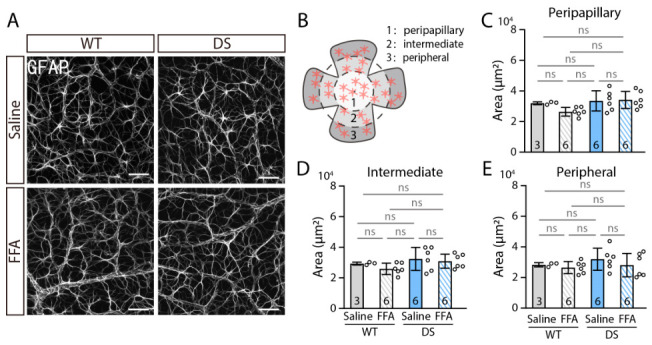
Astrocytes in the retinal nerve fiber layer-ganglion cell layer (NFL-GCL) showed no significant differences between WT and *Scn1a*^+/−^ (DS) groups. (**A**) Representative immunofluorescence labeling of glial fibrillary acidic protein (GFAP) showing retinal astrocytes in the NFL-GCL of WT and DS rats. Images show astrocyte distribution and processes in retinal cross-sections. Scale bar = 50 µm. (**B**) Schematic illustration of the regional segmentation used for NFL-GCL astrocyte analysis: peripapillary (PP), intermediate (IR), and peripheral (PR) regions. (**C**–**E**) Quantification of total GFAP+ astrocyte area in the peripapillary (**C**), intermediate (**H**), and peripheral (**E**) regions. Data are presented as Mean ± SD, per group animals: WT + saline n = 3, WT + FFA n = 6, DS + saline n = 6, and DS + FFA n = 6. Statistical analysis by two-way ANOVA with Tukey’s multiple comparisons test revealed no significant differences between WT and DS groups across all regions: WT + Saline vs. DS + Saline *p* = 0.8142; WT + FFA vs. DS + Saline *p* = 0.1323; DS + Saline vs. DS + FFA *p* = 0.9957 (**C**). WT + Saline vs. DS + Saline *p* = 0.2564; WT + FFA vs. DS + Saline *p* = 0.0852; DS + Saline vs. DS + FFA *p* = 0.9279 (**D**). WT + Saline vs. DS + Saline *p* = 0.0919; WT + FFA vs. DS + Saline *p* = 0.1569; DS + Saline vs. DS + FFA *p* = 0.4046 (**E**). Significance levels: non-significant (ns).

Previous studies in mouse models of Dravet syndrome have reported astrocytic overactivation [10]. To assess whether similar changes occur in our rat model, we examined astrocytes in the nerve fiber layer-ganglion cell layer (NFL-GCL) using glial fibrillary acidic protein (GFAP), an intermediate filament protein widely used as an astrocyte marker. Confocal imaging revealed that GFAP-positive astrocytes form a continuous, interconnected network across the retina (Figure 6A).

We quantified the total area covered by GFAP immunoreactivity in three distinct retinal regions (Figure 6B). In contrast to the significant pathological alterations observed in microglia, we found no significant differences in GFAP coverage area between *Scn1a*^+/−^ and WT rats, nor between saline- and FFA-treated groups. These findings indicate that GFAP-expressing astrocyte density and morphology remain comparable across genotypes and treatments in all examined retina regions (Figure 6C–E), suggesting an absence of reactive astrogliosis in the NFL-GCL of *Scn1a*^+/−^ rats.

### 2.6. Abnormal Retinal Vasculature in Scn1a^+/−^ Rats Is Not Rescued by FFA Treatment

**Figure A5:**
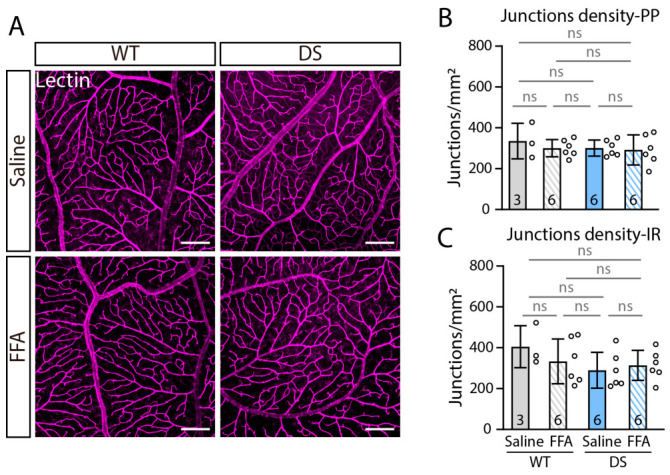
*Scn1a*^+/−^ (DS) rats show abnormalities in blood vessels compared to WT rats, fenfluramine (FFA) treatment did not rescue blood vessel morphology. (**A**) Representative labeling of retinal blood vessels by Tomato lectin. Scale bar = 200 µm. (**B**,**C**) Quantification of vascular branch density (junction density) in the peripapillary (**B**) and intermediate (**C**) regions. Data are presented as Mean ± SD, per group: WT + saline n = 3, WT + FFA n = 6, DS + saline n = 6, and DS + FFA n = 6. Two-way ANOVA: WT + Saline vs. DS + Saline *p* = 0.7215; WT + FFA vs. DS + Saline *p* > 0.9999; DS + Saline vs. DS + FFA *p* = 0.9835 (**B**). WT + Saline vs. DS + Saline *p* = 0.4742; WT + FFA vs. DS + Saline *p* = 0.7536; DS + Saline vs. DS + FFA *p* = 0.9469 (**C**). Significance levels: non-significant (ns).

Astrocytes in the NFL-GCL are closely associated with retinal blood vessels. Moreover, reduced oscillatory potential amplitudes in electroretinography (ERG) are commonly observed in retinopathies involving neurovascular coupling dysfunction, such as diabetic retinopathy [38]. Therefore, we investigated the retinal vasculature network using tomato lectin staining, which preferentially labels endothelial cells in rodent vessels [39]. Confocal imaging revealed comparable vasculature morphology between Dravet syndrome (DS) and wild-type (WT) groups, irrespective of treatment (Figure A5A).

**Figure 7:**
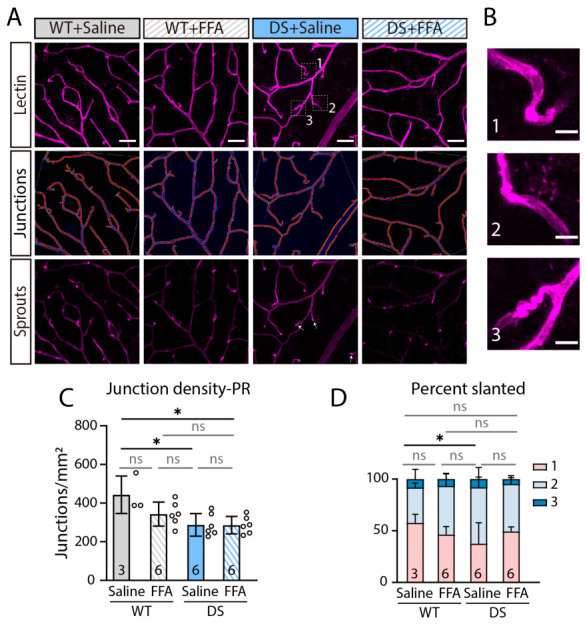
*Scn1a*^+/−^ (DS) rats show abnormalities in blood vessels compared to WT rats, fenfluramine (FFA) treatment did not rescue blood vessel morphology. (**A**) Tomato lectin labeled retinal vasculature. The penetrating vessels in the retina of the DS saline-treated group show slanted (arrows). Scale bar = 50 µm. (**B**) Schematic diagram of slanted vessels: 1 indicates a single slanted, 2 indicates a double slanted, and 3 indicates a triple slanted vessel. Scale bar = 10 µm. (**C**) Quantification of vascular branch density (junction density) in the peripheral regions (PR). Data are presented as Mean ± SD, per group: WT + saline n = 3, WT + FFA n = 6, DS + saline n = 6, and DS + FFA n = 6. Two-way ANOVA: WT + Saline vs. DS + Saline *p* = 0.0135; WT + Saline vs. DS + FFA *p* = 0.0127; DS + Saline vs. DS + FFA *p* > 0.9999. (**D**) Percentage of single slanted (1, red), double slanted (2, light blue), and triple slanted (3, dark blue) vessels. Data are presented as Mean ± SD, per group: WT + saline n = 3, WT + FFA n = 6, DS + saline n = 6, and DS + FFA n = 6. Two-way ANOVA: WT + saline vs. DS + saline *p* = 0.0478 (1); WT + saline vs. DS + saline *p* = 0.0455 (2). Significance levels: *p* < 0.05 (*), non-significant (ns).

Vascular branching form junctions whose density reflects retinal topological organization. We quantified junction density across three retinal regions: peripapillary (PP), intermediate (IR), and peripheral (PR). In peripheral regions, junction density was significantly reduced in *Scn1a*^+/−^ rats treated with either saline or FFA compared to WT rats (Figure 7C). In contrast, junction density remained comparable across genotypes and treatments in both PP and IR regions (Figure A5B,C). These findings indicate that vascular branching topology is compromised in the mutant retina periphery and that FFA treatment does not rescue this phenotype.

During development, retinal blood vessels invade from the inner layer near the retinal ganglion cells and form sprouts as they penetrate outward through the retinal layers [40]. Under physiological conditions, these penetrating vessels predominantly form vertical sprouts orthogonal to the retinal surface (Figure 7A,B-1)). However, in *Scn1a*^+/−^ retinas, we observed an increased proportion of slanted sprouts (Figure 7A, arrows; B-2, B-3). Quantitative analysis confirmed a significant increase in slanted sprouts and a corresponding decrease in vertical sprouts in *Scn1a* mutants, with FFA treatment failing to reverse this shift (Figure 7D).

Collectively, these data demonstrate that the morphological abnormalities in *Scn1a* mutant retinal vasculature persist despite FFA treatment.

## 3. Discussion

Dravet syndrome (DS) is a severe developmental and epileptic encephalopathy (DEE) characterized by a high prevalence of photosensitive seizures [41]. Despite the retina being an integral part of the central nervous system, a comprehensive characterization of retinal disease manifestations in DS has been lacking. The present study reveals significant structural and functional abnormalities in the retina of a *Scn1a*^+/−^ rat model of DS, establishing the retina as a potential window for the diagnosis and prognosis of this neurological disorder.

### 3.1. Retinal Structural Alterations

Utilizing the *Scn1a*^+/−^ rat model of DS [19], we report, for the first time, significant structural alterations in the retina, most notably thinning of the retinal nerve fiber layer (NFL). This observation is consistent with clinical findings in epilepsy patients in general [36,42] and in those with genetic generalized epilepsy in particular [43]. NFL thinning is not restricted to epilepsy; it has emerged as a sensitive biomarker for neurodegenerative diseases and is strongly associated with an increased risk of cognitive decline. In addition to NFL thinning, we identified a concomitant thickening of the outer plexiform layer (OPL), a region containing synaptic connections between photoreceptors and bipolar and horizontal cells. Although OPL alterations have not previously been associated with epilepsy, they have been documented in ischemic retinas of patients with diabetic retinopathy [44]. Collectively, these structural changes in the retina of DS rats suggest the involvement of neurodegenerative and neurovascular pathophysiological processes affecting both inner and outer retinal compartments.

### 3.2. Functional Impairment and Vascular Pathology

Consistent with these gross anatomical changes, we recorded significantly reduced amplitudes of oscillatory potentials (OPs) in DS rats, without alterations in the a-wave or b-wave. Oscillatory potentials-fast oscillatory waveforms superimposed on the ascending limb of the b-wave-represent a more sensitive indicator of retinal function than the b-wave alone. The generation of OPs has been attributed to reciprocal synaptic interactions between bipolar and amacrine cells [45]. Reduced OP amplitudes have been reported in diabetic retinopathy and correlated with the extent of capillary occlusion and increased capillary permeability [46]. Although further investigation is required to elucidate why OPs are particularly vulnerable to alterations in retinal circulation, it is notable that other diseases affecting retinal microcirculation similarly diminish OP amplitudes [46].

In parallel with these functional deficits, DS rats exhibited aberrant vascular morphology, characterized by increased angular curvature of penetrating vessels and reduced branching density. These features resemble phenotypes observed following genetic disruption of vascular patterning molecules [40], suggesting potential dysregulation of angiogenic signaling during development in DS. An impaired blood–brain barrier (BBB) has previously been reported in a mouse model of DS [47]. If retinal microvascular changes correlate with those in the brain, assessment of retinal vasculature by optical coherence tomography angiography (OCTA) could serve as a non-invasive surrogate biomarker for evaluating cerebral vascular integrity.

### 3.3. Neuroinflammatory Responses: Microglia and Astrocytes

Consistent with microglial activation observed in the brains of zebrafish and mouse models of DS [18,48], we detected microglial activation in the retina of DS rats, manifesting as somatic hypertrophy and process retraction—morphological hallmarks of the activated state of microglia [49]. Our findings are also in line with previous reports of microglial activation in the retina of a mouse model of DS [10]. Although prior studies have suggested impaired phagocytosis by microglia in mouse models of DS, the overall observation of microglial activation in both the retina and brain across preclinical models of DS is consistent across species.

In contrast to the consistent microglial phenotype, astrocytes in the retina of rat and mouse models of DS demonstrate divergent phenotypes. We observed an absence of astrogliosis in the retina of the DS rat model, whereas Salazar and colleagues reported astrocyte activation in the retina of a mouse model of DS [10]. A recent report on long-term astrocytic remodeling offers a plausible explanation for this apparent discrepancy [50]. In that study, GFAP+ astrocytes in the hippocampus and cortex remained comparable to controls at the presymptomatic stage but exhibited elevated GFAP levels during disease aggravation and long-term stabilization. This temporal pattern was attributed, in part, to increased astrocyte network connectivity resulting from heightened expression of gap junction proteins during disease progression [50]. Further studies sampling retinas at multiple developmental time points in rats will be essential to address the temporal dynamics of microglial and astrocytic responses in DS.

### 3.4. Therapeutic Implications of Fenfluramine

Fenfluramine (FFA) is a repurposed anorectic agent that has demonstrated effective anti-seizure activity in clinical trials and open-label studies [13,14,15,16,17,51,52]. In mouse models of DS, FFA has been shown to reduce microglial activation, attenuate myelin damage, and promote survival [18]. In the present study, we report that FFA rescued microglial activation in the retina. However, the rescue effect was not accompanied by improvement in retinal vascular architecture, including reduced vessel curvature and vessel branching point density, indicating only a partial rescue effect in total. These findings suggest that FFA effectively modulates microglial activation, potentially through an anti-inflammatory effect [18,53]. Microglia are highly dynamic immune cells in the CNS, their morphological changes suggest a functional shift [54] and indirectly reflect a reduced inflammation in the retina. However, on the contrary, blood vessels invade the retina early in the postnatal development of rodents [40]. FFA treatment starting on P25 may well miss the critical period of angiogenesis and thus fail to completely rescue the retinal microvasculature morphology. An even earlier intervention of FFA may be required for the complete normalization of the vascular deficits. In line with this, advancing FFA treatment for children under 2 years of age is under clinical trials aiming to improve treatment options for young DS patients [55].

### 3.5. Clinical Translation and Future Directions

The findings presented herein support the application of retinal imaging and electroretinography (ERG) as non-invasive biomarkers for monitoring disease progression and therapeutic response in DS. OCT-based retinal analysis offers a safe, rapid, and cost-effective approach that provides high-resolution structural data [56], while ERG supplies complementary functional readouts. Given that retinal screening has already been proposed as an initial screening modality for Alzheimer’s disease [57,58], we envision that retinal morphological and functional assessments may, in the near future, provide valuable prognostic information for DS as well.

In DS, treatment with occlusive patch therapy or blue-tinted lenses has been shown to drastically decrease seizure frequency in some photosensitive patients [59,60]. Although the causal mechanisms linking reduced *Scn1a* expression to photosensitivity remain incompletely understood, the structural and functional abnormalities described in this study represent a first step toward elucidating the relationship between *Scn1a* haploinsufficiency and retinal pathology. Future investigations employing single-cell RNA sequencing, electrophysiological recordings of retinal neurons, and visual evoked potentials (VEPs) may further illuminate the etiology of seizure photosensitivity in DS.

### 3.6. Limitations

We acknowledge that this study has several limitations. First, the sample sizes in each experimental group were relatively small, which may limit statistical power and the generalizability of negative findings. Second, the treatment duration was limited to one week; we did not evaluate the long-term effects of fenfluramine on retinal structure or function, nor did we investigate whether earlier initiation of treatment could yield improved outcomes. Third, fenfluramine is currently believed to exert its antiepileptic effects primarily by promoting the release of serotonin (5-HT) in the central nervous system and inhibiting its reuptake, or via sigma-1 receptors [61,62]; however, the mechanisms underlying FFA’s anti-inflammatory and protective effects on the retina require further investigation. Fourth, although photosensitivity is commonly observed in patients with Dravet syndrome, we did not directly measure light-induced seizure thresholds or behavioral responses to photic stimulation in rats; therefore, the functional correlation between the observed retinal alterations and photosensitivity remains speculative. Fifth, the present study is based on a preclinical model of DS; therefore, further clinical studies in DS patients are required before these findings can be generalized to humans. Finally, our findings support the potential of retinal imaging as a noninvasive biomarker. Future studies with larger sample sizes, longitudinal designs, and clinical validation are needed.

## 4. Materials and Methods

### 4.1. Animals and Genotyping

Wild-type female Sprague-Dawley rats were obtained from Charles River Laboratories (Guangdong, China). All animals were housed under specific pathogen-free (SPF) conditions with food and water available ad libitum. The animal facility maintained a 12 h light-dark cycle (lights on at 8:00 AM, off at 8:00 PM). All procedures were performed in accordance with the institutional animal care guidelines (Protocol Nr: SIAT-IACUC-221020-NS-ROBERT NAUMANN-A2201 20 July 2021, Shenzhen, China), and efforts were made to minimize animal distress and reduce the number of animals used.

*Scn1a* haploinsufficiency (*Scn1a*^+/−^) rats were generated by disrupting exon 20 in the *Scn1a* gene using iGONAD technology [19]. *Scn1a*^+/−^ male rats were bred with WT female rats to produce heterozygous offspring. Offspring were genotyped at P14 via tail biopsy and PCR analysis. Throughout the experiments, littermate rats were used as controls, with each study typically requiring one to two litters. Genotype and sex were randomly assigned within each litter, and both male and female rats were included to ensure a sex-balanced analysis. Rats with body weights markedly deviating from the mean were excluded during the experiments. Based on clinical trial data, interspecies dose conversion factors between rats and humans, and lifespan correspondence [13,63,64,65], we determined the final fenfluramine (FFA) dosing regimen as 5 mg/kg administered daily from P25–P32 for one week. All animal sample processing details are summarized in Table A1.

Genotyping was performed by polymerase chain reaction (PCR) amplification of genomic DNA extracted from tail biopsy tissue as published before [19]. Genomic DNA was isolated using the MouseGenomic DNA Extraction Kit (AD501, TransGen Biotech, Beijing, China), according to the manufacturer’s instructions. PCR amplification was carried out using FastPfu SuperMix (AS221, TransGen Biotech, Beijing, China). Amplification reactions were performed with sequence-specific primers designed to flank the exon 20 in the *Scn1a* gene. Primer sequences are as follows: Forward: 5′–TAGAGTAACAAAACAATAATAACACTGCATGCC–3′, Reverse: 5′–AAGTAATAAAAGTCCTAACTGCACTACGAG–3′, Thermal cycling conditions: initial denaturation at 95 °C for 5 min; followed by 35 cycles of 95 °C for 30 s, 61 °C for 30 s, and 72 °C for 20 s; final extension at 72 °C for 5 min. PCR products were verified by Sanger sequencing with sequencing primer 5′–TAGAGTAACAAAACAATAATAACACTGCATGCC–3′ to determine the genotype.

### 4.2. Electroretinography (ERG)

Visual function was assessed by full-field electroretinography (ERG) using the OPTO-III system (Optoprobe, Pontypridd, UK) [66]. All recordings were performed following overnight dark adaptation (>12 h). Experiments were conducted under dim red light to preserve retinal sensitivity.

Rats (P29) were anesthetized with 3–4% isoflurane in oxygen and maintained on 1.5–2.5% during recording (Animal Anesthesia Device, RWD, Shenzhen, China). Pupils were dilated with 0.5% compound tropicamide eye drops (H20103127, Changchun DiRui Pharmaceutical, Changchun, China), and corneal anesthesia was achieved with 0.5% procaine hydrochloride eye drops (Alcaine, Alcon Pharmaceuticals, Puurs, Belgium). A ring electrode was placed on the corneal surface of each eye, with reference and ground electrodes inserted subcutaneously into the cheek and tail, respectively. Body temperature was monitored and maintained around 37 °C using a homeothermic heating pad.

For scotopic (dark-adapted) maximal mixed response (known as a-wave and b-wave) recordings, stimuli consisted of white light flashes (intensity of 600.0 cd·m^−2^, energy of 3.0 cd·s·m^−2^, flash duration 5 ms) delivered every 15 s. Signals were amplified (×4000), bandpass filtered (1–300 Hz), and averaged over 8 sweeps per trial. Oscillatory potentials (OPs) were recorded under identical stimulus conditions but with an expanded high-frequency filter setting (75–500 Hz) to enhance resolution of high-frequency waves. OP amplitudes were quantified as the sum of the first four peaks (OP1–OP4) measured from trough to peak. Rod response was recorded under dark-adapted conditions, stimuli consisted of white light flashes (intensity of 3.0 cd·m^−2^, energy of 0.015 cd·s·m^−2^, flash duration 5 ms) delivered every 2 s. Signals were also amplified (×4000) and bandpass filtered (1–75 Hz). Light-adapted responses included the cone response and the flicker response. For the cone response, the white background intensity was set to 600.0 cd·m^−2^, with a flash intensity of 3.0 cd·s·m^−2^, bandpass filtered (1–100 Hz). The flicker response was recorded under the same background and flash parameters, except for a stimulation frequency of 20.0 Hz. All data were acquired and analyzed using the integrated visual electrophysiology software (Optoprobe, Pontypridd, UK).

### 4.3. Paraffin Embedding of Eye Specimens and H&E Staining

Paraffin embedding and histological staining were performed as previously described [67]. In brief, P30 rats were transcardially perfused with ice-cold PBS followed by 4% paraformaldehyde (PFA). Eyes were enucleated and initially fixed in Davidson’s fixative at room temperature for 24 h, followed by post-fixation in 4% PFA for an additional 24 h. After fixation, tissues were rinsed thoroughly with PBS.

Under a stereomicroscope, three standardized incisions (“three-window” technique) were made in each eyecup to facilitate proper embedding [67]. The tissues were then dehydrated through a graded ethanol series (30%, 50%, 70%, and 100%), cleared in isopropanol, and embedded in paraffin at 62 °C for 3 h.

Serial sections of 5 µm thickness were cut using a microtome (Leica, Wetzlar, Germany). Sections were floated on a 42 °C water bath for flattening, mounted onto glass slides, and dried overnight at 37 °C to ensure firm adhesion. Slides were stored at room temperature until further processing.

For histological evaluation, sections were deparaffinized, rehydrated, and stained with H&E according to standard protocols. Finally, slides were dehydrated, cleared, and coverslipped for microscopic examination.

### 4.4. Immunofluorescence

Rats were transcardially perfused with ice-cold phosphate-buffered saline (PBS, 0.1 M, pH = 7.4), followed by 4% PFA in PBS. Eyes were carefully enucleated without mechanical compression and post-fixed in fresh 4% PFA at 4 °C for 2 h. Under a stereomicroscope, the cornea and lens were removed, and the retina was gently dissected from the optic cup.

To prepare flat mounts, retinas were cryoprotected by immersion in 30% sucrose in PBS until they sank, then flattened using cold methanol drops applied to the tissue surface to minimize folding. Flat-mounted retinas were stored at −20 °C until use.

For immunofluorescence staining, frozen retinas were thawed and washed three times (10 min each) in PBS. Tissues were blocked and permeabilized in blocking solution containing 1% bovine serum albumin (BSA) and 0.5% Triton X-100 in PBS (PBST) for 2 h at room temperature.

For immunolabeling, the left retina was incubated with Rabbit Polyclonal Anti-Iba1 (1:500, PA5-21274, Invitrogen, Carlsbad, CA, USA) to label microglia. The right retina was incubated with Mouse Monoclonal Anti-GFAP (1:500, #3670s, Cell Signaling Technology, Danvers, MA, USA) and *Lycopersicon esculentum* (Tomato) Lectin (1:200, L32472, Invitrogen, Carlsbad, CA, USA) to label astrocytes and blood vessels, respectively. Retinas were incubated in primary antibody solutions at 4 °C for 48 h with gentle agitation. After three washes in PBST, they were incubated overnight at 4 °C in the dark with appropriate secondary antibodies diluted in PBST: Goat Anti-Rabbit Alexa Fluor 488 (1:1000, A32731, Invitrogen, Carlsbad, CA, USA) or Donkey Anti-Mouse Cy3 (1:1000, 715165150, Jackson ImmunoResearch, West Grove, PA, USA). Following six washes in PBST (30 min total, protected from light), retinas were carefully transferred onto glass slides with the retinal RGC layer facing upward and coverslipped using aqueous mounting medium (F4680, Sigma-Aldrich, St. Louis, MO, USA).

### 4.5. Image Acquisition and Quantitative Analysis

H&E-stained paraffin sections were imaged using an Olympus VS200 slide scanner (Olympus, Tokyo, Japan) equipped with a 20× objective under brightfield illumination. Retinal layer thicknesses were quantitatively assessed using Image J 1.54P software (National Institutes of Health, Bethesda, MD, USA). Measurements were taken at three standardized distances from the optic nerve head: 500 µm, 1000 µm, and 1500 µm. Six evenly distributed fields of view—three on each side of the optic nerve—were analyzed per section. Five serial sections per animal were evaluated to ensure representative sampling.

For whole-mount retinal imaging, a laser scanning confocal microscope (Olympus FV3000, Olympus, Tokyo, Japan) was used. The center of the retina was defined as the origin. Regions of interest were designated based on radial distance from the origin: Peripapillary region (1000–1300 µm), Intermediate region (2000–2300 µm), and Peripheral region (3000–3300 µm).

For Iba1 immunostaining, the outer plexiform layer (OPL) and inner plexiform layer (IPL) were imaged using a 40× objective. Z-stack images were acquired to capture the full depth of microglial morphology. Each sample included 24 fields of view. For Tomato Lectin and GFAP staining, 12 fields of view per sample were captured using either 10× or 40× objectives, with Z-stacks collected to enable three-dimensional reconstruction.

All fluorescence images used for comparative analysis were acquired under identical laser power, gain, and exposure settings to ensure consistency and minimize bias in signal intensity comparisons.

Vascular morphology was analyzed using AngioTool64 0.6a software (NIH, Bethesda, MD, USA), which quantified parameters such as vessel density, branching points, and junctions. Astrocyte coverage area was measured in three regional fields of view (peripapillary, intermediate, and peripheral) using ImageJ 1.8.0, with statistical analysis performed separately for each region.

For morphometric analysis of Iba1-positive microglia, three morphologically intact cells were randomly selected per field of view. Using ImageJ 1.8.0, the cell body area and the total area encompassed by the farthest extensions of cellular processes (i.e., convex hull area) were measured for each cell [10,68]. Data were analyzed independently for each retinal layer and anatomical region.

### 4.6. Statistical Analysis

Statistical analyses were performed using GraphPad Prism 10.1.2. Normality of data distribution was assessed using the Shapiro–Wilk test. For comparisons between two independent groups, an unpaired two-tailed *t*-test was used for normally distributed data; otherwise, the non-parametric Mann–Whitney U test was applied. For data influenced by two independent variables, two-way ANOVA was performed, followed by Tukey’s multiple comparison test to compare specific group means.

All quantitative results are expressed as mean ± standard deviation (SD). Statistical significance is defined as: * *p* < 0.05, ** *p* < 0.01, *** *p* < 0.001, and **** *p* < 0.0001. Data labeled “ns” indicate no statistically significant difference (*p* ≥ 0.05) between groups. All statistical analyses are summarized in the Table A2.

## 5. Conclusions

In this study, we demonstrate significant retinal alterations in a *Scn1a*^+/−^ rat model of Dravet syndrome (DS), including thinning of the nerve fiber layer (NFL), thickening of the outer plexiform layer (OPL), reduced oscillatory potential amplitudes, aberrant vascular morphology, and microglial activation in the OPL and inner plexiform layer (IPL). Fenfluramine (FFA) robustly attenuated microglial activation but failed to rescue gross structural or vascular deficits, indicating a dissociation between its anti-inflammatory efficacy and disease-modifying potential. These findings establish the retina as a promising non-invasive biomarker platform for assessing disease progression and therapeutic response in DS, with multi-modal retinal assessments offering a practical approach for longitudinal clinical monitoring.

## Data Availability

The original contributions presented in this study are included in the article. Further inquiries can be directed to the corresponding authors.

## Abbreviations

The following abbreviations are used in this manuscript:

DS	Dravet syndrome
ERG	Electroretinography
DEE	Developmental and Epileptic Encephalopathy
SUDEP	Sudden Unexpected Death in Epilepsy
FFA	Fenfluramine
EEG	Electroencephalogram
CNS	Central Nervous System
NFL	Nerve Fiber Layer
OPL	Outer Plexiform Layer
GCL	Ganglion Cell Layer
PP	Peripapillary
IR	Intermediate
PR	Peripheral
OPs	Oscillatory Potentials
RGC	Retinal Ganglion Cell
IPL	Inner Plexiform Layer
BBB	Blood–Brain Barrier
INL	Inner Nuclear Layer
ONL	Outer Nuclear Layer
PL	Photoreceptor Layer

## Appendix B

**Table A1:** Summary of animal experiments.

Experiment	Left Eye or Right Eye	Animal Age	Number
Retinal morphology	Left	P30	WT n = 6; DS n = 6
ERG	Left	P29	WT n = 9; DS n = 12
FFA-Retinal morphology	Left	P32	WT n = 4; DS n = 4
FFA-Iba1	Left	P32	WT + saline n = 5
			WT + FFA n = 6
			DS + saline n = 6
			DS + FFA n = 5
FFA-GFAP + lectin	Right	P32	WT + saline n = 3
			WT + FFA n = 6
			DS + saline n = 6
			DS + FFA n = 6

**Table A2:** Summary of all statistical analyses.

Figure	Name	Testing Methods	*p*-Value
Figure 1D	500 μm-NFL	Unpaired *t*-test	0.0181
	500 μm-OPL	Unpaired *t*-test	0.0157
Figure 1F	1000 μm-NFL	Unpaired *t*-test	0.0359
	1000 μm-OPL	Unpaired *t*-test	0.0021
Figure 1H	1500 μm-NFL	Unpaired *t*-test	0.6535
	1500 μm-OPL	Unpaired *t*-test	0.0091
Figure 2C	Max-a	Mann–Whitney test	0.7021
Figure 2D	Max-b	Unpaired *t*-test	0.4565
Figure 2F	OPS	Unpaired *t*-test	0.0196
Figure 3C	500 μm-NFL	Unpaired *t*-test	0.7951
Figure 3D	500 μm-OPL	Unpaired *t*-test	0.1584
Figure 4D IPL-PP-Cell area	WT + Saline vs. WT + FFA	1. ANOVA *p* = 0.0040 2. Tukey’s multiple comparison test	0.2921
	WT + Saline vs. DS + Saline		0.1435
	WT + Saline vs. DS + FFA		0.7847
	WT + FFA vs. DS + Saline		0.003
	WT + FFA vs. DS + FFA		0.806
	DS + Saline vs. DS + FFA		0.0268
Figure 4E IPL-IR-Cell area	WT + Saline vs. WT + FFA	1. ANOVA *p* < 0.0001 2. Tukey’s multiple comparison test	0.0288
	WT + Saline vs. DS + Saline		0.0103
	WT + Saline vs. DS + FFA		0.2719
	WT + FFA vs. DS + Saline		<0.0001
	WT + FFA vs. DS + FFA		0.5829
	DS + Saline vs. DS + FFA		0.0003
Figure 5C OPL-PP-Intensity	WT + saline vs. WT + FFA	1. ANOVA *p* = 0.0107 2. Tukey’s multiple comparison test	0.9665
	WT + saline vs. DS + saline		0.0295
	WT + saline vs. DS + FFA		0.2053
	WT + FFA vs. DS + saline		0.015
	WT + FFA vs. DS + FFA		0.106
	DS + saline vs. DS + FFA		0.5787
Figure 5D OPL-IR-Intensity	WT + saline vs. WT + FFA	1. ANOVA *p* = 0.0004 2. Tukey’s multiple comparison test	0.7954
	WT + saline vs. DS + saline		0.0013
	WT + saline vs. DS + FFA		0.391
	WT + FFA vs. DS + saline		0.0004
	WT + FFA vs. DS + FFA		0.1108
	DS + saline vs. DS + FFA		0.0122
Figure 5E OPL-PP-Cell area	WT + Saline vs. WT + FFA	1. ANOVA *p* = 0.0023 2. Tukey’s multiple comparison test	0.9629
	WT + Saline vs. DS + Saline		0.0041
	WT + Saline vs. DS + FFA		0.8788
	WT + FFA vs. DS + Saline		0.0059
	WT + FFA vs. DS + FFA		0.9919
	DS + Saline vs. DS + FFA		0.0164
Figure 5F OPL-IR-Cell area	WT + Saline vs. WT + FFA	1. ANOVA *p* = 0.0108 2. Tukey’s multiple comparison test	0.999
	WT + Saline vs. DS + Saline		0.025
	WT + Saline vs. DS + FFA		0.9984
	WT + FFA vs. DS + Saline		0.0211
	WT + FFA vs. DS + FFA		>0.9999
	DS + Saline vs. DS + FFA		0.0337
Figure 5G OPL-PP-Arbor area	WT + Saline vs. WT + FFA	1. ANOVA *p* = 0.0087 2. Tukey’s multiple comparison test	0.9521
	WT + Saline vs. DS + Saline		0.0448
	WT + Saline vs. DS + FFA		0.9933
	WT + FFA vs. DS + Saline		0.0109
	WT + FFA vs. DS + FFA		0.9939
	DS + Saline vs. DS + FFA		0.0276
Figure 5G OPL-IR-Arbor area	WT + Saline vs. WT + FFA	1. ANOVA *p* = 0.0002 2. Tukey’s multiple comparison test	0.9977
	WT + Saline vs. DS + Saline		0.0015
	WT + Saline vs. DS + FFA		0.967
	WT + FFA vs. DS + Saline		0.0006
	WT + FFA vs. DS + FFA		0.9917
	DS + Saline vs. DS + FFA		0.0007
Figure 5G OPL-PR-Arbor area	WT + Saline vs. WT + FFA	1. ANOVA *p* = 0.0001 2. Tukey’s multiple comparison test	0.785
	WT + Saline vs. DS + Saline		0.0003
	WT + Saline vs. DS + FFA		0.966
	WT + FFA vs. DS + Saline		0.0008
	WT + FFA vs. DS + FFA		0.9651
	DS + Saline vs. DS + FFA		0.0006
Figure 6C Peripapillary	WT + Saline vs. WT + FFA	1. ANOVA *p* = 0.0574 2. Tukey’s multiple comparison test	0.7693
	WT + Saline vs. DS + Saline		0.8142
	WT + Saline vs. DS + FFA		0.7203
	WT + FFA vs. DS + Saline		0.1323
	WT + FFA vs. DS + FFA		0.0911
	DS + Saline vs. DS + FFA		0.9957
Figure 6D Intermediate	WT + Saline vs. WT + FFA	1. ANOVA *p* = 0.2042 2. Tukey’s multiple comparison test	0.9996
	WT + Saline vs. DS + Saline		0.2564
	WT + Saline vs. DS + FFA		0.4739
	WT + FFA vs. DS + Saline		0.0852
	WT + FFA vs. DS + FFA		0.2243
	DS + Saline vs. DS + FFA		0.9279
Figure 6E Peripheral	WT + Saline vs. WT + FFA	1. ANOVA *p* = 0.4828 2. Tukey’s multiple comparison test	0.8331
	WT + Saline vs. DS + Saline		0.0919
	WT + Saline vs. DS + FFA		0.5475
	WT + FFA vs. DS + Saline		0.1569
	WT + FFA vs. DS + FFA		0.909
	DS + Saline vs. DS + FFA		0.4046
Figure 7C junction density-PR	WT + Saline vs. WT + FFA	1. ANOVA *p* = 0.0106 2. Tukey’s multiple comparison test	0.1343
	WT + Saline vs. DS + Saline		0.0135
	WT + Saline vs. DS + FFA		0.0127
	WT + FFA vs. DS + Saline		0.3512
	WT + FFA vs. DS + FFA		0.3316
	DS + Saline vs. DS + FFA		>0.9999
Figure 7D Percent in slanted-1	WT + saline vs. WT + FFA	1. ANOVA *p* < 0.0001 2. Tukey’s multiple comparison test	0.4398
	WT + saline vs. DS + saline		0.0478
	WT + saline vs. DS + FFA		0.6851
	WT + FFA vs. DS + saline		0.4879
	WT + FFA vs. DS + FFA		0.9613
	DS + saline vs. DS + FFA		0.2339
Figure 7D Percent in slanted-2	WT + saline vs. WT + FFA	1. ANOVA *p* < 0.0001 2. Tukey’s multiple comparison test	0.3344
	WT + saline vs. DS + saline		0.0455
	WT + saline vs. DS + FFA		0.4078
	WT + FFA vs. DS + saline		0.6178
	WT + FFA vs. DS + FFA		0.9984
	DS + saline vs. DS + FFA		0.5144
Figure 7D Percent in slanted-3	WT + saline vs. WT + FFA	1. ANOVA *p* < 0.0001 2. Tukey’s multiple comparison test	0.9975
	WT + saline vs. DS + saline		>0.9999
	WT + saline vs. DS + FFA		0.9685
	WT + FFA vs. DS + saline		0.9967
	WT + FFA vs. DS + FFA		0.9883
	DS + saline vs. DS + FFA		0.951

## References

[1] Balestrini S., Scheffer I.E. (2026). Ameliorating Seizures in Dravet Syndrome: A Review of Newly Approved and Investigational Drugs, RNA and Gene-Based Therapies. CNS Drugs.

[2] Dravet C. (2011). The core Dravet syndrome phenotype. Epilepsia.

[3] Tro-Baumann B., von Spiczak S., Lotte J., Bast T., Haberlandt E., Sassen R., Freund A., Leiz S., Stephani U., Boor R. (2011). A retrospective study of the relation between vaccination and occurrence of seizures in Dravet syndrome. Epilepsia.

[4] Verbeek N.E., van der Maas N.A., Sonsma A.C., Ippel E., Vermeer-de Bondt P.E., Hagebeuk E., Jansen F.E., Geesink H.H., Braun K.P., de Louw A. (2015). Effect of vaccinations on seizure risk and disease course in Dravet syndrome. Neurology.

[5] Rosch R.E., Goldberg E.M., Baram T.Z., Shinnar S., Stafstrom C.E. (2023). Chapter 3—SCN1A and Dravet syndrome. Febrile Seizures.

[6] Valassina N., Brusco S., Salamone A., Serra L., Luoni M., Giannelli S., Bido S., Massimino L., Ungaro F., Mazzara P.G. (2022). Scn1a gene reactivation after symptom onset rescues pathological phenotypes in a mouse model of Dravet syndrome. Nat. Commun..

[7] Ricci D., Porto C., Massaroni V., Chieffo D.P.R., Battaglia D.I. (2026). Early neurovisual development in Dravet syndrome. Neurosci. Biobehav. Rev..

[8] Chieffo D., Ricci D., Baranello G., Martinelli D., Veredice C., Lettori D., Battaglia D., Dravet C., Mercuri E., Guzzetta F. (2011). Early development in Dravet syndrome; visual function impairment precedes cognitive decline. Epilepsy Res..

[9] Ceulemans B., Schoonjans A.-S., Van de Vel A., Kasteleijn-Nolst Trenite D. (2021). Photosensitivity in Dravet Syndrome. The Importance of Photosensitivity for Epilepsy.

[10] Salazar J.J., Satriano A., Matamoros J.A., Fernández-Albarral J.A., Salobrar-García E., López-Cuenca I., de Hoz R., Sánchez-Puebla L., Ramírez J.M., Alonso C. (2023). Retinal Tissue Shows Glial Changes in a Dravet Syndrome Knock-in Mouse Model. Int. J. Mol. Sci..

[11] Boncristiano A., Balestrini S., Doccini V., Specchio N., Pietrafusa N., Trivisano M., Darra F., Cossu A., Battaglia D., Quintiliani M. (2025). Fenfluramine treatment for Dravet syndrome: Long term real-world analysis demonstrates safety and reduced health care burden. Epilepsia.

[12] Xia D., Zhang P., Chen Y., Liu X., Chen Y. (2024). Efficacy of pharmacological treatments for Dravet syndrome: Systematic review and network meta-analysis. Seizure.

[13] Lagae L., Sullivan J., Knupp K., Laux L., Polster T., Nikanorova M., Devinsky O., Cross J.H., Guerrini R., Talwar D. (2019). Fenfluramine hydrochloride for the treatment of seizures in Dravet syndrome: A randomised, double-blind, placebo-controlled trial. Lancet.

[14] Scheffer I.E., Nabbout R., Lagae L., Devinsky O., Auvin S., Thiele E.A., Wirrell E.C., Polster T., Specchio N., Pringsheim M. (2025). Long-term safety and effectiveness of fenfluramine in children and adults with Dravet syndrome. Epilepsia.

[15] Bishop K.I., Isquith P.K., Gioia G.A., Gammaitoni A.R., Farfel G., Galer B.S., Nabbout R., Wirrell E.C., Polster T., Sullivan J. (2021). Improved everyday executive functioning following profound reduction in seizure frequency with fenfluramine: Analysis from a phase 3 long-term extension study in children/young adults with Dravet syndrome. Epilepsy Behav..

[16] Sullivan J., Lagae L., Cross J.H., Devinsky O., Guerrini R., Knupp K.G., Laux L., Nikanorova M., Polster T., Talwar D. (2023). Fenfluramine in the treatment of Dravet syndrome: Results of a third randomized, placebo-controlled clinical trial. Epilepsia.

[17] Nabbout R., Mistry A., Zuberi S., Villeneuve N., Gil-Nagel A., Sanchez-Carpintero R., Stephani U., Laux L., Wirrell E., Knupp K. (2020). Fenfluramine for Treatment-Resistant Seizures in Patients with Dravet Syndrome Receiving Stiripentol-Inclusive Regimens: A Randomized Clinical Trial. JAMA Neurol..

[18] Cha J., Filatov G., Smith S.J., Gammaitoni A.R., Lothe A., Reeder T. (2024). Fenfluramine increases survival and reduces markers of neurodegeneration in a mouse model of Dravet syndrome. Epilepsia Open.

[19] Li M., Yang L., Qian W., Ray S., Lu Z., Liu T., Zou Y.Y., Naumann R.K., Wang H. (2023). A novel rat model of Dravet syndrome recapitulates clinical hallmarks. Neurobiol. Dis..

[20] Nestler E.J., Hyman S.E. (2010). Animal models of neuropsychiatric disorders. Nat. Neurosci..

[21] Sinn R., Wittbrodt J. (2013). An eye on eye development. Mech. Dev..

[22] London A., Benhar I., Schwartz M. (2013). The retina as a window to the brain—From eye research to CNS disorders. Nat. Rev. Neurol..

[23] Chiquita S., Rodrigues-Neves A.C., Baptista F.I., Carecho R., Moreira P.I., Castelo-Branco M., Ambrósio A.F. (2019). The Retina as a Window or Mirror of the Brain Changes Detected in Alzheimer’s Disease: Critical Aspects to Unravel. Mol. Neurobiol..

[24] Kirbas S., Turkyilmaz K., Anlar O., Tufekci A., Durmus M. (2013). Retinal nerve fiber layer thickness in patients with Alzheimer disease. J. Neuroophthalmol..

[25] Masland R.H. (2001). The fundamental plan of the retina. Nat. Neurosci..

[26] Grimes W.N., Berson D.M., Sabnis A., Hoon M., Sinha R., Tian H., Diamond J.S. (2025). Layer-specific anatomical and physiological features of the retina’s neurovascular unit. Curr. Biol..

[27] Masland R.H. (2012). The neuronal organization of the retina. Neuron.

[28] Gelatt K.N. (2014). Optics and Physiology of Vision. Essentials of Veterinary Ophthalmology.

[29] Newman E.A., Zahs K.R. (1998). Modulation of neuronal activity by glial cells in the retina. J. Neurosci..

[30] Karlstetter M., Ebert S., Langmann T. (2010). Microglia in the healthy and degenerating retina: Insights from novel mouse models. Immunobiology.

[31] Santos A.M., Calvente R., Tassi M., Carrasco M.C., Martín-Oliva D., Marín-Teva J.L., Navascués J., Cuadros M.A. (2008). Embryonic and postnatal development of microglial cells in the mouse retina. J. Comp. Neurol..

[32] Rathnasamy G., Foulds W.S., Ling E.A., Kaur C. (2019). Retinal microglia—A key player in healthy and diseased retina. Prog. Neurobiol..

[33] Silverman S.M., Wong W.T. (2018). Microglia in the Retina: Roles in Development, Maturity, and Disease. Annu. Rev. Vis. Sci..

[34] Zhao D., Pinares-Garcia P., McKenzie C.E., Bleakley L.E., Forster I.C., Wong V.H.Y., Nguyen C.T.O., Scheffer I.E., Reid C.A., Bui B.V. (2023). Retinal Dysfunction in a Mouse Model of HCN1 Genetic Epilepsy. J. Neurosci..

[35] Liu P.K., Huang W.C., Wang N.K., Tsang S.H., Quinn P.M. (2023). Electroretinogram (ERG) to Evaluate the Retina Using Mouse Models. Retinitis Pigmentosa.

[36] Balestrini S., Clayton L.M., Bartmann A.P., Chinthapalli K., Novy J., Coppola A., Wandschneider B., Stern W.M., Acheson J., Bell G.S. (2016). Retinal nerve fibre layer thinning is associated with drug resistance in epilepsy. J. Neurol. Neurosurg. Psychiatry.

[37] Midena E., Torresin T., Longhin E., Midena G., Pilotto E., Frizziero L. (2021). Early Microvascular and Oscillatory Potentials Changes in Human Diabetic Retina: Amacrine Cells and the Intraretinal Neurovascular Crosstalk. J. Clin. Med..

[38] Shinoda K., Rejdak R., Schuettauf F., Blatsios G., Völker M., Tanimoto N., Olcay T., Gekeler F., Lehaci C., Naskar R. (2007). Early electroretinographic features of streptozotocin-induced diabetic retinopathy. Clin. Exp. Ophthalmol..

[39] Robertson R.T., Levine S.T., Haynes S.M., Gutierrez P., Baratta J.L., Tan Z., Longmuir K.J. (2015). Use of labeled tomato lectin for imaging vasculature structures. Histochem. Cell Biol..

[40] Toma K., Zhao M., Zhang S., Wang F., Graham H.K., Zou J., Modgil S., Shang W.H., Tsai N.Y., Cai Z. (2024). Perivascular neurons instruct 3D vascular lattice formation via neurovascular contact. Cell.

[41] Verbeek N., Kasteleijn-Nolst Trenité D., Wassenaar M., van Campen J., Sonsma A., Gunning W.B., de Weerd A., Knoers N., Spetgens W., Gutter T. (2017). Photosensitivity in Dravet syndrome is under-recognized and related to prognosis. Clin. Neurophysiol..

[42] Stauner L., Bao H., Delazer L., Kirsch I., Christmann T., Noachtar S., Havla J., Lauseker M., Kaufmann E. (2024). Longitudinal evaluation of retinal neuroaxonal loss in epilepsy using optical coherence tomography. Epilepsia.

[43] González de la Aleja J., Guerrero-Molina M., Saíz-Díaz R.A., López-Muñoz F., Raga-Martínez I., Hernández-Gallego J., Navarrete-Chamorro P., Povedano-Montero F.J. (2019). Peripapillary retinal nerve fibre layer thinning in genetic generalized epilepsy. Seizure.

[44] Reznicek L., Kernt M., Haritoglou C., Kampik A., Ulbig M., Neubauer A.S. (2010). In vivo characterization of ischemic retina in diabetic retinopathy. Clin. Ophthalmol..

[45] Liao F., Liu H., Milla-Navarro S., Villa P., Germain F. (2023). Origin of Retinal Oscillatory Potentials in the Mouse, a Tool to Specifically Locate Retinal Damage. Int. J. Mol. Sci..

[46] McAnany J.J., Persidina O.S., Park J.C. (2022). Clinical electroretinography in diabetic retinopathy: A review. Surv. Ophthalmol..

[47] Alonso C., García-Culebras A., Satta V., Hernández-Fisac I., Sierra Á., Guimaré J.A., Lizasoain I., Fernández-Ruiz J., Sagredo O. (2025). Investigation in blood-brain barrier integrity and susceptibility to immune cell penetration in a mouse model of Dravet syndrome. Brain Behav. Immun. Health.

[48] Brenet A., Somkhit J., Csaba Z., Ciura S., Kabashi E., Yanicostas C., Soussi-Yanicostas N. (2024). Microglia Mitigate Neuronal Activation in a Zebrafish Model of Dravet Syndrome. Cells.

[49] Davis E.J., Foster T.D., Thomas W.E. (1994). Cellular forms and functions of brain microglia. Brain Res. Bull..

[50] Genin A., Janvier A., Moujellil-Legagneur T., Blaquière M., Chaussy A., Privé R., Duprat F., Mantegazza M., Audinat E., Marchi N. (2026). Long-lasting remodeling of astrocytes in an Scna1(+/−) mouse model of Dravet syndrome. Epilepsia.

[51] Polster T. (2019). Individualized treatment approaches: Fenfluramine, a novel antiepileptic medication for the treatment of seizures in Dravet syndrome. Epilepsy Behav..

[52] Schoonjans A., Paelinck B.P., Marchau F., Gunning B., Gammaitoni A., Galer B.S., Lagae L., Ceulemans B. (2017). Low-dose fenfluramine significantly reduces seizure frequency in Dravet syndrome: A prospective study of a new cohort of patients. Eur. J. Neurol..

[53] Wiens K.R., Brooks N.A.H., Riar I., Greuel B.K., Lindhout I.A., Klegeris A. (2024). Psilocin, the Psychoactive Metabolite of Psilocybin, Modulates Select Neuroimmune Functions of Microglial Cells in a 5-HT(2) Receptor-Dependent Manner. Molecules.

[54] Paolicelli R.C., Sierra A., Stevens B., Tremblay M.E., Aguzzi A., Ajami B., Amit I., Audinat E., Bechmann I., Bennett M. (2022). Microglia states and nomenclature: A field at its crossroads. Neuron.

[55] Pietrafusa N., Trivisano M., Casellato S., Correale C., Cappelletti S., De Liso P., Onida I., Sotgiu S., Butera A., Specchio N. (2024). Fenfluramine below the age of 2 years in Dravet syndrome: What about safety and efficacy?. Epilepsia.

[56] Nolan R.C., Narayana K., Galetta S.L., Balcer L.J. (2015). Optical Coherence Tomography for the Neurologist. Semin. Neurol..

[57] Zhang Y., Wang Y., Shi C., Shen M., Lu F. (2021). Advances in retina imaging as potential biomarkers for early diagnosis of Alzheimer’s disease. Transl. Neurodegener..

[58] Alber J., Bouwman F., den Haan J., Rissman R.A., De Groef L., Koronyo-Hamaoui M., Lengyel I., Thal D.R. (2024). Retina pathology as a target for biomarkers for Alzheimer’s disease: Current status, ophthalmopathological background, challenges, and future directions. Alzheimers Dement..

[59] Choi C., Khuddus N., Mickler C., Tuli S., Tuli S. (2011). Occlusive patch therapy for reduction of seizures in Dravet syndrome. Clin. Pediatr..

[60] Takahashi J.S. (1995). Molecular neurobiology and genetics of circadian rhythms in mammals. Annu. Rev. Neurosci..

[61] Sourbron J., Smolders I., de Witte P., Lagae L. (2017). Pharmacological Analysis of the Anti-epileptic Mechanisms of Fenfluramine in scn1a Mutant Zebrafish. Front. Pharmacol..

[62] Martin P., Reeder T., Sourbron J., de Witte P.A.M., Gammaitoni A.R., Galer B.S. (2021). An Emerging Role for Sigma-1 Receptors in the Treatment of Developmental and Epileptic Encephalopathies. Int. J. Mol. Sci..

[63] Nair A.B., Jacob S. (2016). A simple practice guide for dose conversion between animals and human. J. Basic. Clin. Pharm..

[64] Andreollo N.A., Santos E.F., Araújo M.R., Lopes L.R. (2012). Rat’s age versus human’s age: What is the relationship?. Arq. Bras. Cir. Dig..

[65] Quinn R. (2005). Comparing rat’s to human’s age: How old is my rat in people years?. Nutrition.

[66] Brandli A., Stone J. (2015). Using the Electroretinogram to Assess Function in the Rodent Retina and the Protective Effects of Remote Limb Ischemic Preconditioning. J. Vis. Exp..

[67] Pang J., Thomas N., Tsuchiya D., Parmely T., Yan D., Xie T., Wang Y. (2021). Step-by-step preparation of mouse eye sections for routine histology, immunofluorescence, and RNA in situ hybridization multiplexing. STAR Protoc..

[68] Rojas P., Ramírez A.I., Cadena M., Fernández-Albarral J.A., Salobrar-García E., López-Cuenca I., Santos-García I., de Lago E., Urcelay-Segura J.L., Ramírez J.M. (2021). Retinal Ganglion Cell Loss and Microglial Activation in a SOD1G93A Mouse Model of Amyotrophic Lateral Sclerosis. Int. J. Mol. Sci..

